# Two new species of the genus *Tsauria* Koçak & Kemal (Hemiptera, Fulgoromorpha, Cixiidae) from China, with descriptions of female genitalia of three species

**DOI:** 10.3897/zookeys.855.34024

**Published:** 2019-06-13

**Authors:** Yan Zhi, Pei Zhang, Lin Yang, Xiang-sheng Chen

**Affiliations:** 1 Institute of Entomology, Guizhou University, Guiyang, Guizhou, 550025, China; 2 The Provincial Special Key Laboratory for Development and Utilization of Insect Resources of Guizhou, Guizhou University, Guiyang, Guizhou, 550025, China; 3 Laboratory Animal Center, Guizhou Medical University, Guiyang, Guizhou 550025, China; 4 Xingyi Normal University for Nationalities, Xingyi, Guizhou, 562400, China

**Keywords:** Female genitalia, Fulgoroidea, morphology, Oriental region, taxonomy

## Abstract

Two new species of cixiid planthoppers genus *Tsauria* Koçak & Kemal, *Tsauriabrevispina* Zhi & Chen, **sp. nov.** and *T.longispina* Zhi & Chen, **sp. nov.**, are described and illustrated from China and *T.transspinus* (Zhang & Chen, 2011) was removed to give the genus four species in total. The female genitalia of three species are described and illustrated for the first time. A key to all known species of *Tsauria* based on male genitalia, and a key to three species (except for *T.major*) based on female genitalia, are provided.

## Introduction

Tsaur et al. (1991) established the cixiid planthopper genus *Discophorellus* with the type species *Discophorellusmajor* Tsaur & Hsu, 1991 from China (Taiwan), and placed this genus in the tribe Cixiini of the subfamily Cixiinae (Hemiptera: Fulgoromorpha: Cixiidae). Later, Koçak and Kemal (2009) proposed a new replacement name *Tsauria* for *Discophorellus* Tsaur & Hsu, 1991 because the latter is a junior homonym of *Discophorellus* Wibmer & O’Brien, 1986 (Coleoptera). But, Zhang and Chen (2011) did not recognize the homonym of *Discophorellus* and described two new species from China: *D.cehengensis* and *D.transspinus*. Subsequently, Xing and Chen (2014) transferred the two species described by Zhang and Chen (2011) to *Tsauria*. However, the taxonomic status of *T.transspinus* (Zhang & Chen, 2011) is reviewed in this study and, based on a few diagnostic characters, removed from *Tsauria* (see Discussion). So far, *Tsauria* includes two species: *T.cehengensis* (Zhang & Chen, 2011) and *T.major* (Tsaur & Hsu, 1991).

Herein, two new species: *Tsauriabrevispina* Zhi & Chen, sp. nov. and *T.longispina* Zhi & Chen, sp. nov. are described and illustrated from China. Female genitalia of three Chinese species are described and illustrated for the first time. The genus now includes four species, and all from China. A key to all known species of *Tsauria* based on male genitalia, and a key to three species (except for *T.major*) based on female genitalia, are provided.

## Materials and methods

The morphological terminology and measurements follow Bourgoin (1987) and Bourgoin et al. (2015). The morphological terminology of female genitalia follows Bourgoin (1993). Body length was measured from apex of vertex to tip of forewing; vertex length was measured the median length of vertex (from apical transverse carina to tip of basal emargination). Fuchsin staining was used to highlight the female genitalia structures studied. Ten to fifteen female specimens per species were dissected. External morphology and drawings were done with the aid of a Leica MZ 12.5 stereomicroscope. Photographs were taken with KEYENCE VHX-1000 system. Illustrations were scanned with CanoScan LiDE 200 and imported into Adobe Photoshop CS7 for labeling and plate composition. The dissected male and female genitalia are preserved in glycerin in small plastic tubes pinned together with the specimens.

The type specimens examined are deposited in the Institute of Entomology, Guizhou University, Guiyang, Guizhou Province, China (GUGC).

## Taxonomy

### Tsauria

Taxon classificationAnimaliaHemipteraCixiidae

Koçak & Kemal, 2009

Discophorellus Tsaur & Hsu, 1991: 21; Zhang and Chen 2011: 60.
Tsauria Koçak & Kemal, 2009: 6 for Discophorellus Tsaur & Hsu, 1991, nec Wibmer & O’Brien, 1986; Xing and Chen 2014: 149.

#### Type species.

*Discophorellusmajor* Tsaur & Hsu, 1991, by original designation. For the relationship and diagnosis of *Tsauria* see Tsaur et al. (1991: 21) and Zhang and Chen (2011: 60).

#### Distribution.

Oriental region (China).

##### Key to species (males) of *Tsauria* (revised from Zhang and Chen 2011)

**Table:** 

1	Ventral margin of aedeagal periandrium with an extremely long spinose process, which is the longest of all spinose processes of periandrium (Figs 46–49)	***T.longispina* sp. nov.**
–	Spinose process on ventral margin of aedeagal periandrium not the longest of periandrium	**2**
2	Ventral margin of aedeagal periandrium with an extremely short spinose process, which is the shortest of all spinose processes of periandrium (Figs 13–16)	***T.brevispina* sp. nov.**
–	Spinose process on ventral margin of aedeagal periandrium not the shortest of periandrium	**3**
3	Forewings with r-m cross-vein and apical cells yellowish brown (Zhang and Chen 2011: Fig. 36); medioventral process of pygofer papillary in ventral view, with bristles at apex (Zhang and Chen 2011: Fig. 5)	*** T. cehengensis ***
–	Forewings with r-m cross-vein and apical cells black; medioventral process of pygofer sub-triangular in ventral view, rounded and smooth at apex (Tsaur et al. 1991: Fig. 10E)	*** T. major ***

##### Key to species (females) of *Tsauria* (except for *T.major*)

**Table:** 

1	Wax plate divided by median keel (Fig. 53)	***T.longispina* sp. nov.**
–	Wax plate widened laterally and without median keel	**2**
2	The length of posterior vagina (Figs 34–35) equal to the width. Sclerites in ventral view mainly concentrated in the middle area and the ones in dorsal view mainly concentrated on left side. Gonapophysis IX (Fig. 32) with two middle teeth, denticulate portion with one small rounded odontoid	*** T. cehengensis ***
–	Posterior vagina (Figs 24–25) elongate, with sclerites dispersed both in ventral and dorsal view. Gonapophysis IX (Fig. 22) with one middle tooth, denticulate portion degenerated	***T.brevispina* sp. nov.**

### Tsauria brevispina

Taxon classificationAnimaliaHemipteraCixiidae

Zhi & Chen sp. nov.

http://zoobank.org/30979941-88CE-413C-9E3F-E4C2C1B1BDD1

Figs 1–2 ; 5–16 , 17–26

#### Type material.

Holotype: ♂, **China**: Hubei, Luotian County, Dabieshan, 15 July 2010, Jun-qiang Ni; paratypes: 3♀♀, Hubei, Luotian County, Dabieshan, 15–17 July 2010, Jun-qiang Ni; 2♂♂5♀♀, Hubei, Luotian County, Dabieshan, Qingtaiguan, 2–3 July 2014, Mei-na Guo, Jian-kun Long, Zheng-xiang Zhou; 1♂2♀♀, Hubei, Luotian County, Dabieshan, Taohuachong, 23–28 June 2014, Mei-na Guo, Hai-yan Sun; 1♂1♀, Hubei, Luotian County, Dabieshan, Wujiashan, 27–29 June 2014, Mei-na Guo, Zheng-xiang Zhou; 5♂♂5♀♀, Guizhou, Tongren, Fanjingshan, Heihewan, 18 May 2013, Wei-cheng Yang, Yu-bo Zhang, Jian-kun Long; 1♂, Guizhou, Guiyang, Huaxi, Qingyan, 20 July 2012, Zhi-hua Fan.

#### Description.

Body length: male 6.9–7.5 mm (*n* = 11), female 7.0–8.8 mm (*n* = 16).

**Figures 1–4.**
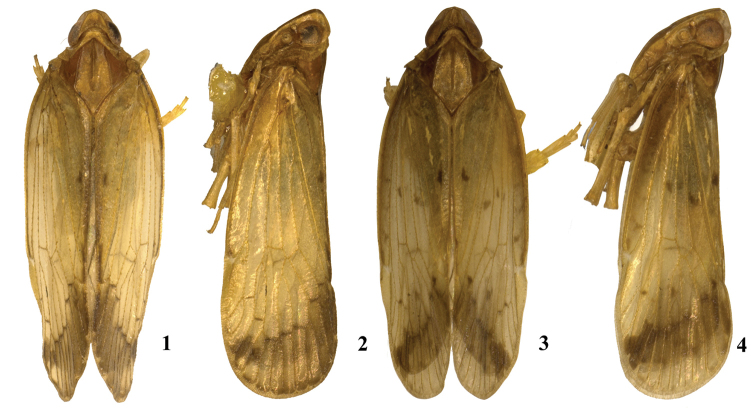
*Tsauriabrevispina* sp. nov., male **1** dorsal view **2** lateral view **3–4***Tsaurialongispina* sp. nov., male **3** dorsal view **4** lateral view.

**Figures 5–16.**
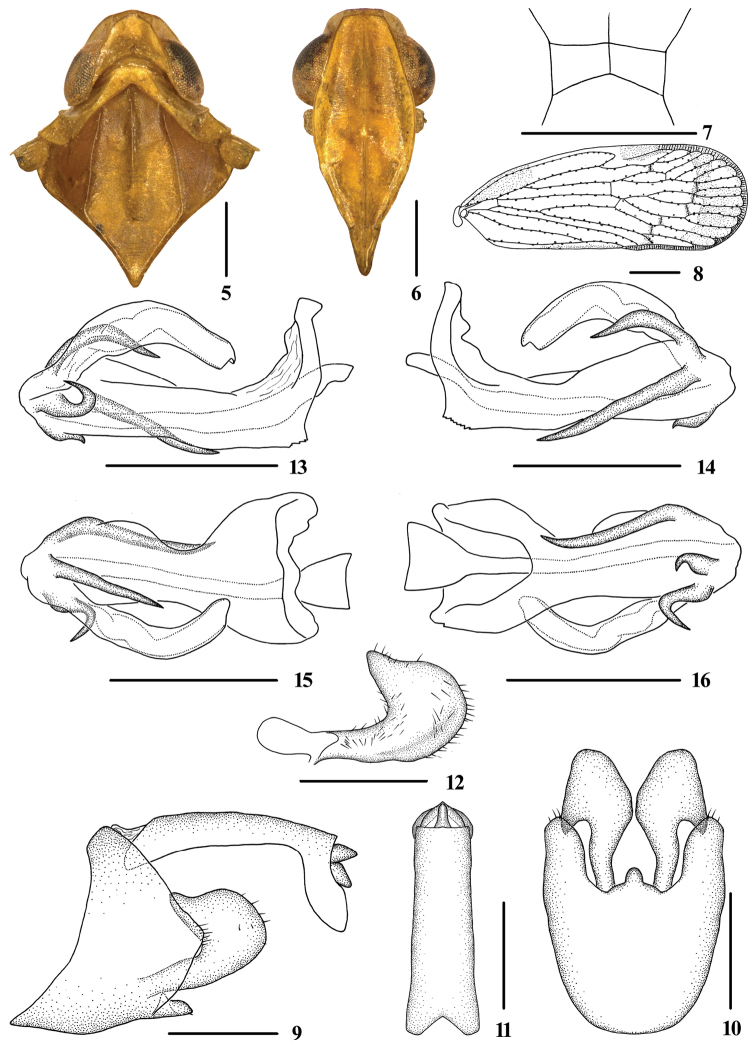
*Tsauriabrevispina* sp. nov., male **5** head and thorax, dorsal view **6** face, ventral view **7** head, top view **8** forewing **9** genitalia, lateral view **10** pygofer and gonostyli, ventral view **11** anal segment, dorsal view **12** gonostyli, inner lateral view **13** aedeagus, right side **14** aedeagus, left side **15** aedeagus, dorsal view **16** aedeagus, ventral view. Scale bars: 0.5 mm (**5–7, 9–16**); 1.0 mm **(8**).

Coloration. General color yellowish brown (Figs 1, 2, 5, 6). Eyes yellowish brown, ocelli yellow. Vertex, face, rostrum and pronotum yellowish brown, mesonotum brown. Forewing semi-translucent, yellowish brown, stigma yellowish brown, termination of forewing blackish brown. Hind tibiae and abdominal sternites yellowish brown.

Head and thorax. Vertex (Figs 1, 5, 7) broad, 1.5 times wider than long; subapical carina with middle prominent into obtuse angle, median carina interrupted by subapical carina, with anterior portion complete, posterior portion only discernible at basal half. Frons (Fig. 6) 1.2 times as long as wide. Clypeus with median carina distinct and elevated throughout. Pronotum (Figs 1, 5) 1.9 times longer than vertex. Mesonotum 1.6 times longer than pronotum and vertex combined. Forewing (Fig. 8) 2.7 times longer than wide, with 13 apical and 7 subapical cells; RP 4 branches, MP with 5 terminals: MP_11_, MP_12_, MP_2_, MP_3_, and MP_4_, fork MP_1_+MP_2_ basad of fork MP_3_+MP_4_. Hind tibia with 3‒5 lateral spines; chaetotaxy of hind tarsi: 8–9/10–11, second segment of hind tarsus with 7 platellae.

Male genitalia. Pygofer (Figs 9, 10) symmetrical, dorsal margin concave and U-shaped ventrally, widened towards apex; in lateral view, lateral lobes triangularly extended caudally. Medioventral process mastoid ventrally. Anal segment (Figs 9, 11) long tubular, symmetrical, 2.5 times longer than wide in dorsal view; anal style finger-like, not beyond anal segment. Gonostyli (Figs 9, 10, 12) in ventral view, symmetrical, widening towards apex, apical part extended, apical margin rounded; in lateral view, “L-shaped”. Aedeagus (Figs 13–16) in total with four processes. Spinose process on left side near apex of periandrium being the longest, straight, directed ventrocephalically; right side of periandrium with a medium-sized spinose process, strongly curved, directed dorsocaudally at apex; periandrium with a medium-sized spinose process positioning slightly to left side of its dorsal margin, directed right-ventrocephalically; ventral margin of aedeagal periandrium with an extremely short spinose process, which is the shortest of all spinose processes of periandrium, hooked, curved towards right side. Endosoma moderately sclerotized, simple, generally curving left.

**Figures 17–26.**
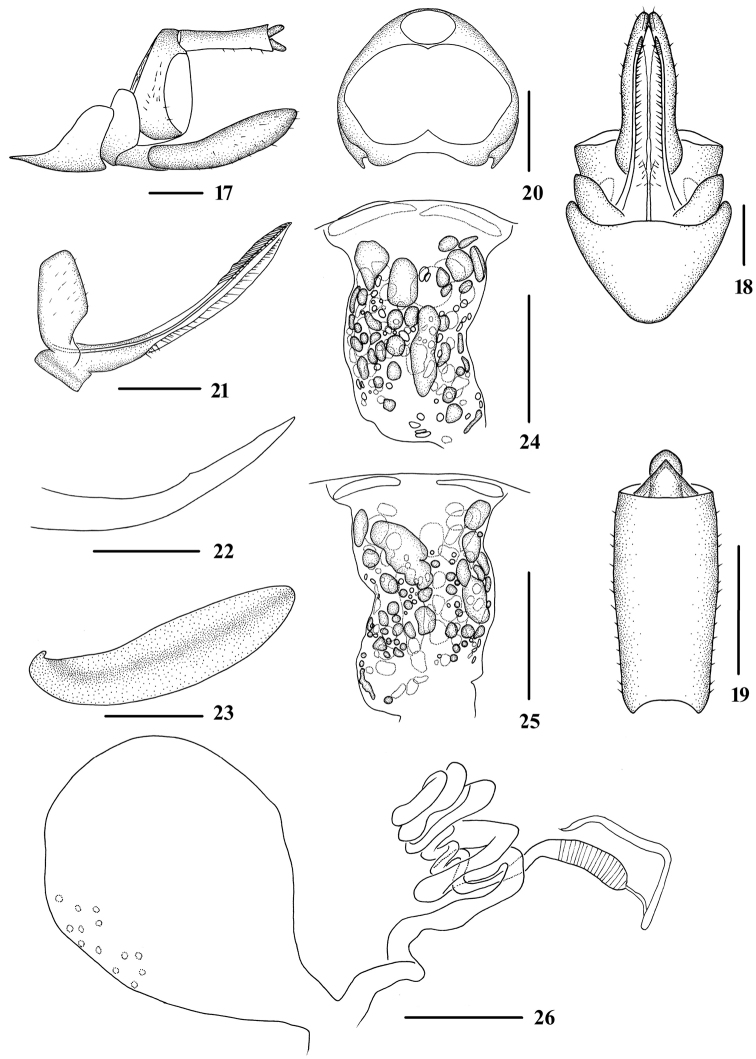
*Tsauriabrevispina* sp. nov., female **17** genitalia, lateral view **18** genitalia, ventral view **19** anal segment, dorsal view **20** tergite IX, caudal view **21** gonapophysis VIII and gonocoxa VIII, dorsal view **22** gonapophysis IX, lateral view **23** gonoplac, inner lateral view **24** posterior vagina, ventral view **25** posterior vagina, dorsal view **26** internal genitalia. Scale bars: 0.5 mm.

Female genitalia. Tergite IX (Figs 17, 18, 20) moderately sclerotized, with a large nearly elliptical wax plate. Anal segment (Figs 17, 19) rectangle, 2.2 times longer than wide in dorsal view. Gonapophysis VIII (Fig. 21) elongate, and slightly curved upwards. Gonapophysis IX (Fig. 22) with one middle tooth, denticulate portion degenerated. Gonoplac (Fig. 23) rod-like, 3.7 times longer than wide in lateral view. Posterior vagina (Figs 24, 25) elongate, with many small round, oval and oblong sclerites both in ventral and dorsal view, dispersed. Base with several relatively large sclerites, and the middle area with a longitudinally oblong sclerite in ventral view; at basal each lateral side with several relatively large sclerites respectively in dorsal view. Internal genitalia as shown in Fig. 26.

#### Distributions.

China (Guizhou, Hubei).

#### Etymology.

The specific name is derived from the Latin prefixes “*brevi*” and noun “*spina*”, referring to the ventral margin of aedeagal periandrium with an extremely short spinose process, which is the shortest of all spinose processes of the periandrium.

#### Remarks.

Male genitalia of *T.brevispina* sp. nov. is similar to *T.cehengensis* (Zhang & Chen), but differs in: (1) spinose process on ventral margin of periandrium being the shortest of all spinose processes of periandrium (in *T.cehengensis*, not the shortest one); (2) spinose process on left side near apex of periandrium being the longest, straight (in *T.cehengensis*, spinose process in the same position being the shortest, basal two-thirds stout and apical third arc-shaped curved); (3) medioventral process without bristles at apex (the latter with bristles); (4) forewing with 13 apical cells (the latter with 12 apical cells).

Female genitalia of *T.brevispina* sp. nov. is similar to *T.cehengensis* (Zhang & Chen), but differs in: (1) posterior vagina elongate (in *T.cehengensis*, the length of posterior vagina equal to the width); (2) sclerites dispersed both in ventral and dorsal view (in *T.cehengensis*, sclerites in ventral view mainly concentrated in the middle area and the ones in dorsal view mainly concentrated in left side); (3) Gonapophysis IX with one middle tooth, denticulate portion degenerated (in *T.cehengensis*, Gonapophysis IX with two middle teeth, denticulate portion with one small rounded odontoid).

### Tsauria cehengensis

Taxon classificationAnimaliaHemipteraCixiidae

(Zhang & Chen, 2011)

Figs 27–37

Discophorellus cehengensis Zhang & Chen, 2011: 61, figs 1–11, 36–37.
Tsauria cehengensis (Zhang & Chen, 2011): Xing and Chen 2014: 149.

#### Material examined.

**China**: 1♂, Guizhou, Ceheng County (900 m), 29 June–1 July 2006, Qiong-zhang Song (holotype); 9♂♂3♀♀, same data as holotype, Qiong-zhang Song, Pei Zhang (paratypes); 4♀♀, Guizhou, Ziyun County, Getuhe, Dahemiaozhai (930 m), 24–27 June 2006, Pei Zhang (paratypes); 2♂♂, Guizhou, Libo County, Maolan, Banzhai, 4–6 July 2010, Pei Zhang, Xiao-hui Hou; 1♂1♀, Guizhou, Libo County, Maolan, Wengang, 4 July 2010, Pei Zhang; 1♂, Guizhou, Huishui County, Duanshan, Guangrong, 9 May 2013, Jian-kun Long; 1♂2♀♀, Guizhou, Guiyang, Forest Park, 25 June 2010, Yan-li Zheng; 1♂1♀, Jiangsu, Longnan County, Jiulianshan, Daqiutian, 24 July 2009, Ze-hong Meng; 3♂♂3♀♀, Anhui, Huangshan City, Tangkou (500 m), 20 May 2008, Zheng-guang Zhang; 17♂♂19♀♀, Guangxi, Shangsi County, Shiwandashan National Forest Park, 2 May 2011, Rong Huang, Xiao-fei Yu; 1♀, Guangxi, Shangsi County, Shiwandashan National Forest Park, 9 June 2012, Jian-kun Long; 1♂1♀, Guangxi, Shangsi County, Shiwandashan National Forest Park, 30 May 2012, Nan-nan Yang; 1♂1♀, Guizhou, Leishan County, Leigongshan, Xiaodanjiang, 6–8 July 2011, Jian-kun Long, Wei-bin Zheng; 1♂2♀♀, Guizhou, Wangmo County, Xintun, 28-June 2013, Jian-kun Long, Yang-yang Liu; 1♂, Guizhou, Congjiang County, Guanghui, 20 July 2016, Zheng-xue Zhao.

#### Supplementary description.

**Figures 27–37.**
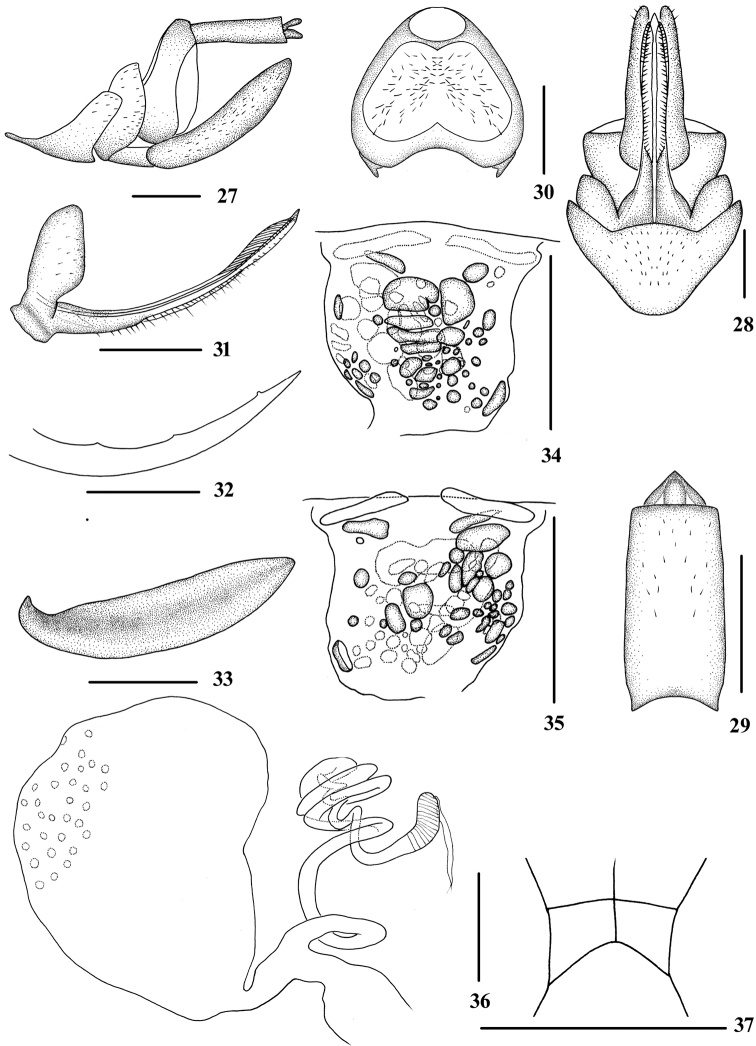
*Tsauriacehengensis* (Zhang & Chen, 2011) **27–36**, female **27** genitalia, lateral view **28** genitalia, ventral view **29** anal segment, dorsal view **30** tergite IX, caudal view **31** gonapophysis VIII and gonocoxa VIII, dorsal view **32** gonapophysis IX, lateral view **33** gonoplac, inner lateral view **34** posterior vagina, ventral view **35** posterior vagina, dorsal view **36** internal genitalia **37** head, top view, male. Scale bars: 0.5 mm.

Female genitalia. Tergite IX (Figs 27, 28, 30) moderately sclerotized, with a large nearly trapezoidal wax plate. Anal segment (Figs 27, 29) rectangle, 2.1 times longer than wide in dorsal view. Gonapophysis VIII (Fig. 31) elongate, and slightly curved upwards. Gonapophysis IX (Fig. 32) with two middle teeth, denticulate portion with only one small rounded odontoid. Gonoplac (Fig. 33) rod-like, 3.9 times longer than wide in lateral view. The length of posterior vagina (Figs 34, 35) equal to the width. Posterior vagina with many small round, oval and oblong sclerites both in ventral and dorsal view. Sclerites in ventral view mainly concentrated in the middle area and the ones in dorsal view mainly concentrated in left side. Internal genitalia as shown in Fig. 36.

#### Distributions.

China (Anhui, Jiangsu, Guangxi, Guizhou).

#### Note.

The female genitalia of this species are described and illustrated for the first time.

### Tsauria longispina

Taxon classificationAnimaliaHemipteraCixiidae

Zhi & Chen sp. nov.

http://zoobank.org/A394C767-D2ED-4689-89DB-5B16D0CF4232

Figs 3 , 4 ; 38–49 , 50–59

#### Type material.

Holotype: ♂, **China**: Zhejiang, Hangzhou City, Tianmushan, 22 July 2009, Ting-ting He; paratypes: 24♂♂33♀♀, Zhejiang, Hangzhou City, Tianmushan, 20–22 July 2009, Yong Chen, Ting-ting He; 1♂, Zhejiang, Longquan City, Fengyangshan, 28–29 July 2009, Ting-ting He; 2♂♂, Guizhou, Liping County, Taipingshan (520–859 m), 15–23 July 2006, Zheng-Guang Zhang; 1♂4♀♀, Guizhou, Liping County, Deshun, 14 July 2016, Yan-li Zheng, Nian Gong, Zheng-xue Zhao, Ying-jian Wang; 1♂, Hainan, Wuzhishan (650 m), 14 July 2007, Ji-chun Xing; 3♂♂, Fujian, Jianou City, Wanmulin, 8–10 August 2009, Pei Zhang, Jun-qiang Ni; 2♂♂4♀♀, Fujian, Jianou City, Wanmulin, 20 May 2012, Jian-kun Long, Wei-cheng Yang; 1♂1♀, Fujian, Dehua county, Guobao, Yunlonggu, 11 May 2012, Jian-kun Long, Wei-cheng Yang; 1♂, Fujian, Datian County, Forest Park, 14 May 2012, Wei-cheng Yang.

#### Description.

Body length: male 6.8–7.6 mm (*n* = 37), female 7.0–8.3 mm (*n* = 42).

**Figures 38–49.**
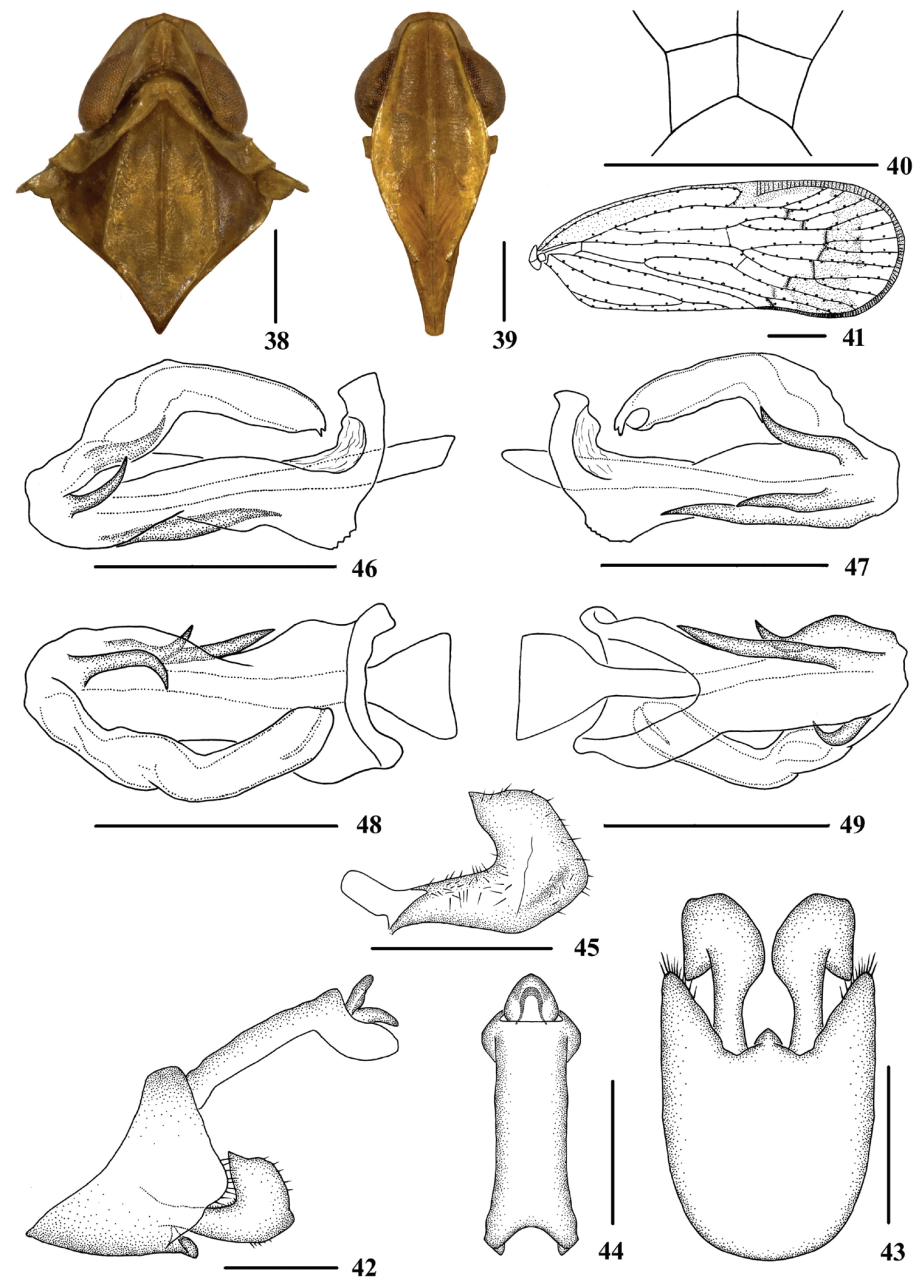
*Tsaurialongispina* sp. nov., male **38** head and thorax, dorsal view **39** face, ventral view **40** head, top view **41** forewing **42** genitalia, lateral view **43** pygofer and gonostyli, ventral view **44** anal segment, dorsal view **45** gonostyli, inner lateral view **46** aedeagus, right side **47** aedeagus, left side **48** aedeagus, dorsal view **49** aedeagus, ventral view. Scale bars: 0.5 mm (**38–40, 42–49**); 1.0 mm **(41**).

Coloration. General color yellowish brown (Figs 3, 4, 38–39). Eyes yellowish brown, ocelli pale yellow. Vertex, face, rostrum, pronotum and mesonotum brown. Forewing semi-translucent, yellowish brown, apical 1/5 with a wide blackish brown stripe, stigma yellowish brown. Hind tibiae yellowish brown and abdominal sternites dark brown.

Head and thorax. Vertex (Figs 3, 38, 40) broad, 1.3 times wider than long; subapical carina with middle prominent into obtuse angle, median carina interrupted by subapical carina, with anterior portion complete, posterior portion only discernible at basal half. Frons (Fig. 39) 1.2 times as long as wide. Clypeus with median carina distinct and elevated throughout. Pronotum (Figs 3, 38) 1.8 times longer than vertex; mesonotum 1.5 times longer than pronotum and vertex combined. Forewing (Fig. 41) 2.7 times longer than wide, with 12 apical and 7 subapical cells; RP 3 branches, MP with 5 terminals: MP_11_, MP_12_, MP_2_, MP_3_, and MP_4_, fork MP_1_+MP_2_ basad of fork MP_3_+MP_4_. Hind tibia with 3‒4 lateral spines; chaetotaxy of hind tarsi: 9/10‒12, second segment of hind tarsus with 6‒9 platellae.

Male genitalia. Pygofer (Figs 42, 43) symmetrical, dorsal margin concave and U-shaped ventrally; in lateral view, lateral lobes triangularly extended caudally, apex round. Medioventral process mastoid ventrally. Anal segment (Figs 42, 44) long tubular, symmetrical, 2.9 times longer than wide in dorsal view; anal style finger-like, slightly beyond anal segment. Gonostyli (Figs 42, 43, 45) in ventral view, symmetrical, widening towards apex, apical part extended, apical margin rounded; in lateral view, “L-shaped”. Aedeagus (Figs 46–49) in total with four processes. Left side of periandrium with a medium-sized spinose process, slightly curved, directed left-ventrocephalically at apex; right side near apex of periandrium with a short spinose process, directed left-dorsocephalically; periandrium with a medium-sized spinose process positioning slightly to left side of its dorsal margin, slightly curved upward and directed right-dorsally at apex; ventral margin of aedeagal periandrium with an extremely long spinose process, which is the longest of all spinose processes of periandrium, straight, generally directed towards left side, apex directed cephalically. Endosoma moderately sclerotized, structure simple, generally curving left.

**Figures 50–59.**
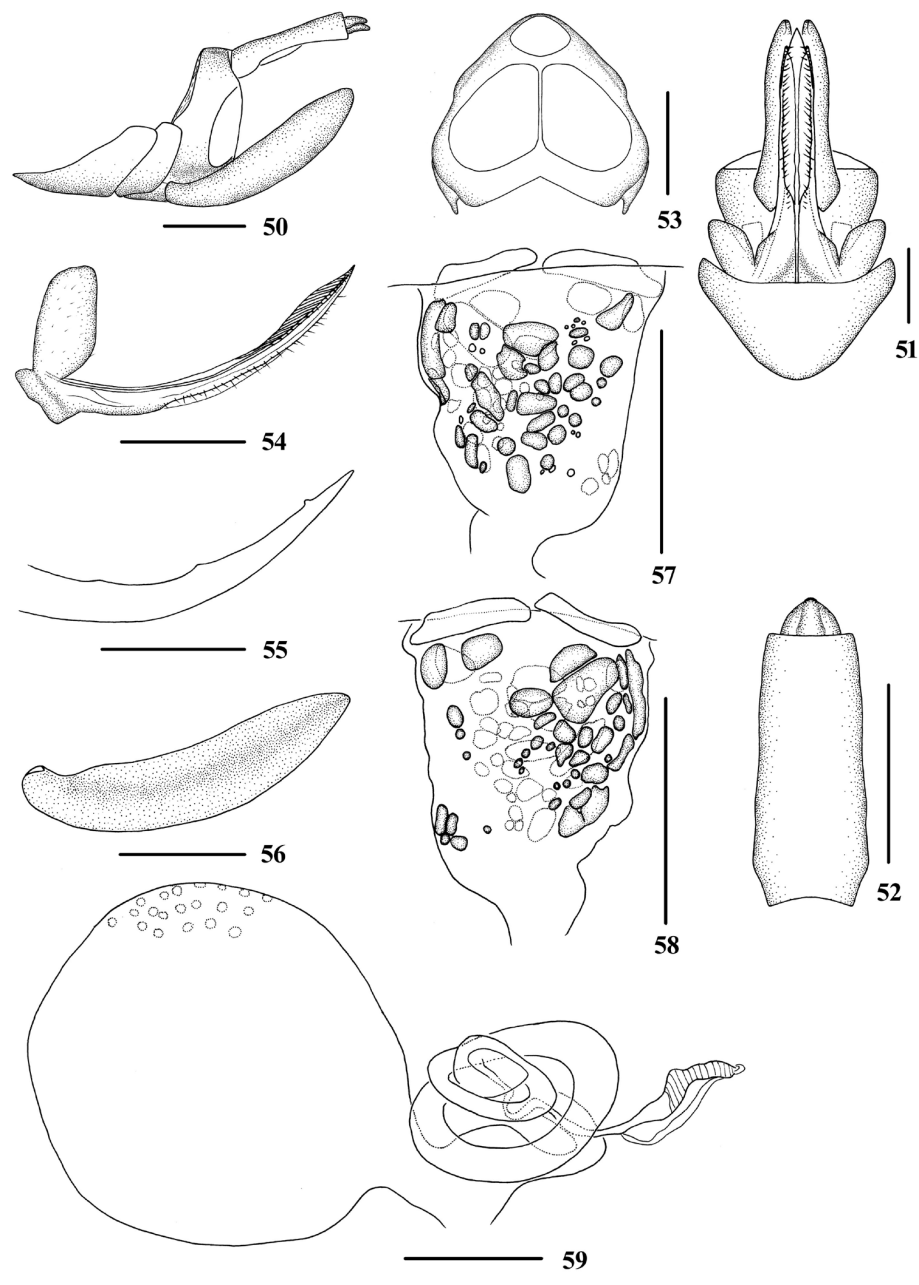
*Tsaurialongispina* sp. nov., female **50** genitalia, lateral view **51** genitalia, ventral view **52** anal segment, dorsal view **53** tergite IX, caudal view **54** gonapophysis VIII and gonocoxa VIII, dorsal view **55** gonapophysis IX, lateral view **56** gonoplac, inner lateral view **57** posterior vagina, ventral view **58** posterior vagina, dorsal view **59** internal genitalia. Scale bars: 0.5 mm.

Female genitalia. Tergite IX (Figs 50, 51, 53) subtriangular, moderately sclerotized, divided by median keel. Anal segment (Figs 50, 53) rectangle, 2.3 times longer than wide in dorsal view. Gonapophysis VIII (Fig. 54) elongate, and slightly curved upwards. Gonapophysis IX (Fig. 55) with two middle teeth, denticulate portion with only one small rounded odontoid. Gonoplac (Fig. 56) rod-like, 3.7 times longer than wide in lateral view. Posterior vagina (Figs 57, 58) elongate. Posterior vagina with many small round, oval and oblong sclerites both in ventral and dorsal view. Sclerites in ventral view dispersed and the ones in dorsal view mainly concentrated in left side. Internal genitalia as shown in Fig. 59.

#### Distributions.

China (Fujian, Guizhou, Hainan, Zhejiang).

#### Etymology.

The specific name is derived from the Latin prefixes “*longi*” and noun “*spina*”, referring to the ventral margin of aedeagal periandrium with an extremely long spinose process, which is the longest of all spinose processes of the periandrium.

#### Remarks.

Male genitalia of *T.longispina* sp. nov. is similar to *T.brevispina* sp. nov., but differs in: (1) spinose process on ventral margin of periandrium being the longest of all spinose processes of periandrium, straight (in *T.brevispina*, spinose process on ventral margin of periandrium being the shortest of all spinose processes of periandrium, hooked at apex); (2) spinose process on right side near apex of periandrium slightly curved, directed left-dorsocephalically at apex (the latter strongly curved, directed dorsocaudally at apex).

Female genitalia of *T.longispina* sp. nov. is similar to *T.cehengensis* (Zhang & Chen), but differs in: (1) wax plate divided by median keel (the latter widened laterally and without median keel; (2) posterior vagina elongate (in *T.cehengensis*, the length of posterior vagina equal to the width).

## Discussion

Holzinger (2002) emphasized the importance of the morphological characters of the female abdomen wax plate and its conformation in Cixiini. The conformation of the wax-plate area below the anal tube in females has great taxonomic value in Cixiidae. Therefore, following Holzinger’s taxonomic practice, we confirmed that the species *Tsauriatransspinus* (Zhang & Chen, 2011) had been incorrectly placed in this genus, for its females lacked the wax plate (Fig. 61), whereas the existence of this structure was critical in *Tsauria*. Some other characters, such as “vertex with median carina before subapical carina vanished (Fig. 63) and abdomen with shorter anal tube in females (Figs 61–62)” were also distinctly inconsistent with the other members of *Tsauria*. For the above reasons, in this study we removed *Tsauriatransspinus* from *Tsauria* and left it as incertae sedis provisionally, as determining its taxonomic position was out of the main purpose of this paper, and it probably should be dealt with elsewhere.

**Figures 60–63.**
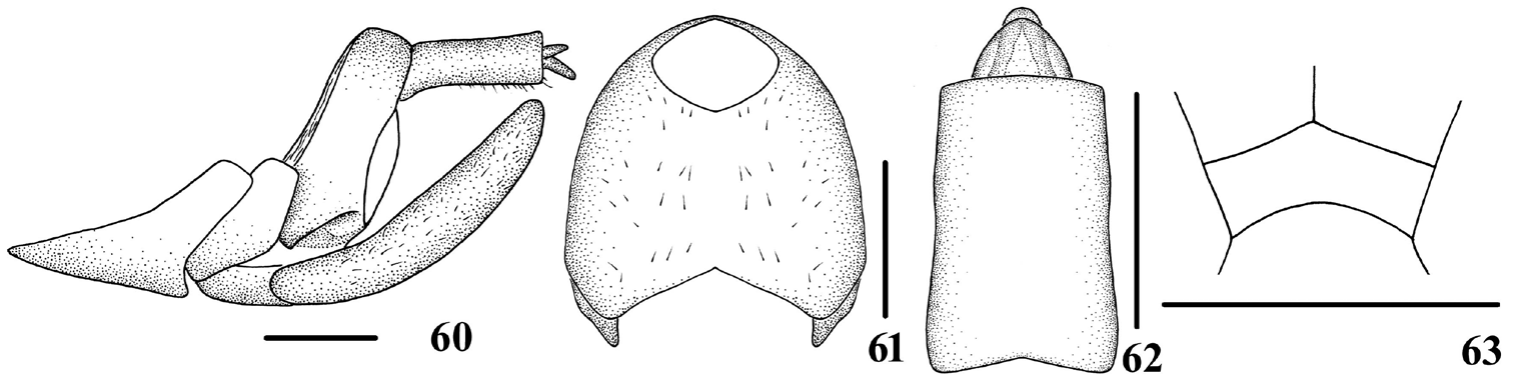
*Tsauriatransspinus* (Zhang & Chen, 2011) incertae sedis, female **60** genitalia, lateral view **61** tergite IX, caudal view **62** anal segment, dorsal view **63** head, top view, male. Scale bars: 0.5 mm.

*Tsauriacehengensis* (Zhang & Chen, 2011) and *T.major* (Tsaur & Hsu, 1991) were distinguished mainly on the characters of the male genitalia; only the anal segment of *T.major* was illustrated for the female (Tsaur et al. 1991; Zhang and Chen 2011). Zhi et al. (2017, 2018) found the characters of the sclerites on the posterior vagina could be considered as key diagnostic characters for female identification in the genera *Neocarpia* (Eucarpiini) and *Oecleopsis* (Pentastirini). The authors also discussed the external and the internal structures of the female genitalia in cixiid planthoppers. In this study, the sclerites of the vagina are studied in detail in *Tsauriabrevispina* (Figs 24, 25), *T.cehengensis* (Figs 34, 35) and *T.longispina* (Figs 57, 58). As a result, the characters of the posterior vagina have been shown to be fairly eﬀective when used to distinguish among species of *Tsauria*.

## Supplementary Material

XML Treatment for Tsauria

XML Treatment for Tsauria brevispina

XML Treatment for Tsauria cehengensis

XML Treatment for Tsauria longispina
